# Supplementary material for: Deep Receptor Scanning Reveals General Sequence Constraints on GPCR Biosynthesis
Source: bioRxiv. 2025 Sep 21:2025.09.19.677468. Preprint. [Version 1] doi: 10.1101/2025.09.19.677468 (PMC12458215; doi:10.1101/2025.09.19.677468)
Supplement: Supplement 1 [file media-1.pdf]

Supplementary Information for:

Deep Receptor Scanning Reveals General Sequence Constraints on GPCR Biosynthesis

Austin Tedman,[1] Muskan Goel,[1] Sohan Shah,[1] Mathew K. Howard,[2,8] Laura M. Chamness,[3] Antonio Bonifasi,[1] Ismalia Adams,[4] Jacklyn M. Gallagher,[1] Wesley D. Penn,[5] Katarina Nemec,[6] Eli F. McDonald,[6] Brianna N. Corman,[7] J. Paul Robinson,[7] Carol B. Post,[4] Patricia Clark,[3] M. Madan Babu,[6] Aashish Manglik,[8] Charles P. Kuntz,[1*] Willow Coyote-Maestas,[2*] and Jonathan P. Schlebach[1*]

[1] The James Tarpo Jr. and Margaret Tarpo Department of Chemistry, Purdue University, West Lafayette, IN, USA
[2] Department of Bioengineering and Therapeutic Science, University of California, San Francisco, San Francisco, CA, USA
[3]Department of Chemistry and Biochemistry, University of Notre Dame, Notre Dame, IN, USA
[4]The Borch Department of Medicinal Chemistry and Molecular Pharmacology, Purdue University, West Lafayette, IN, USA
[5]Department of Chemistry, Indiana University, Bloomington, IN, USA
[6]Center of Excellence for Data Driven Discovery, St. Jude Children's Research Hospital, Memphis, TN, USA
[7]Weldon School of Biomedical Engineering, Purdue University, West Lafayette, IN, USA
[8]Department of Pharmaceutical Chemistry, University of California, San Francisco, San Francisco, CA, USA

*Corresponding Authors: jschleba (at) purdue.edu, willow.coyote-maestas (at) ucsf.edu, and cpkuntz (at) purdue.edu

Contents:

-Figure S1
-Figure S2
-Figure S3
-Figure S4
-Table S1
-Table S2
-Table S3

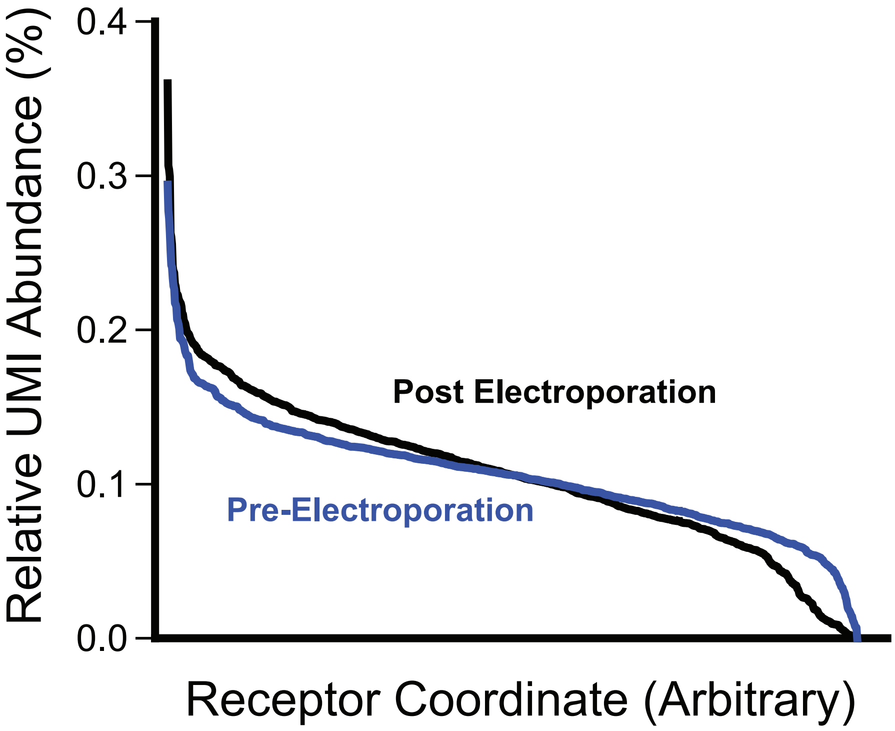

Figure S1. Relative Abundance of GPCR Plasmids within Pooled Libraries. 946 individual plasmid preparations were hand-mixed at an equal stoichiometric ratio prior to electroporation of the pool into *E. coli*. The plasmid pool was then purified from *E. coli* prior to comparison of the relative abundance of each GPCR-associated UMI in the initial pool and the final plasmid preparation by deep sequencing. Individual UMIs are plotted onto an arbitrary coordinate according to the percentage of the total reads withn each plasmid population. Overall, the variance between individual UMIs is typically modest and is remains similar after transformation into *E. coli*.

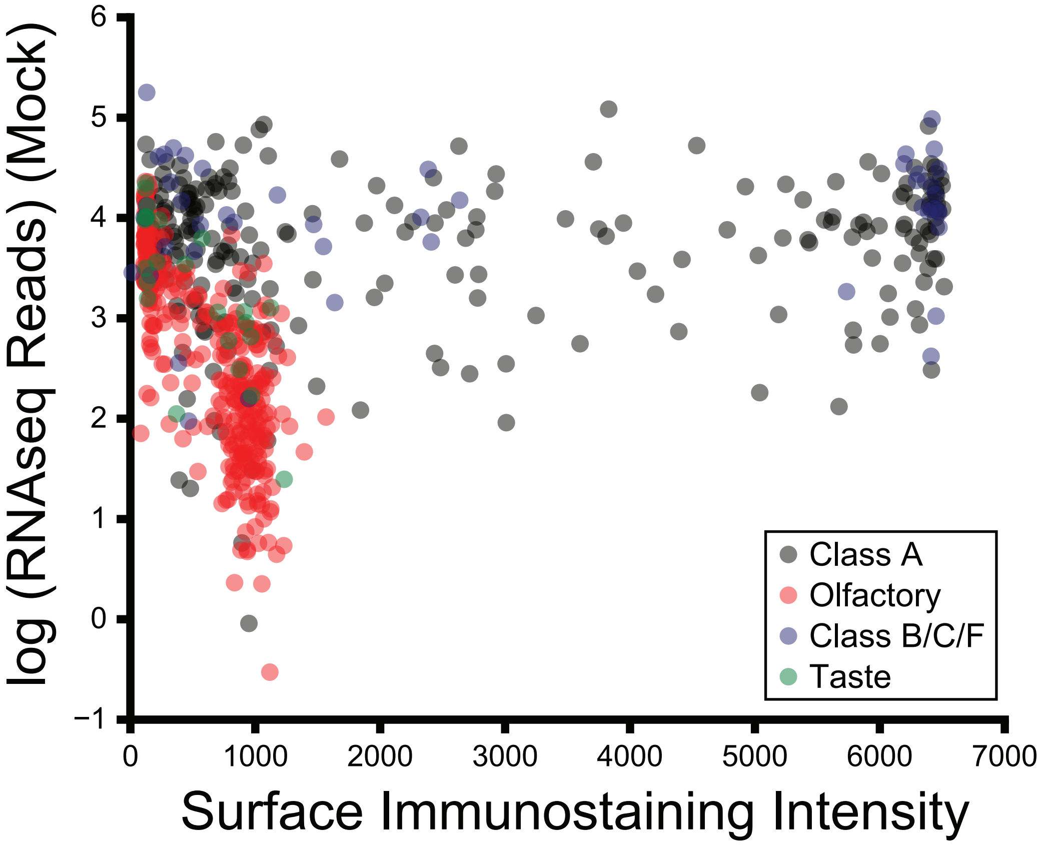

Figure S2. GPCR Transcript Abundance and Surface Expression. A scatterplot depicts the $\log_{10}$ transformation of the number reads per kilobase for each GPCR-encoding transcript relative to the plasma membrane expression of the corresponding receptor protein. The number pf transcript reads for each receptor within a mock transfection sample were subtracted from the number of reads for the corresponding transcript within a pool of recombinant cells expressing GPCRs in order to correct for any endogenous receptor transcripts, which should not influence the immunostaining of the HA-tagged receptors. Transcripts are colored according to the class of the receptor they encode, for reference

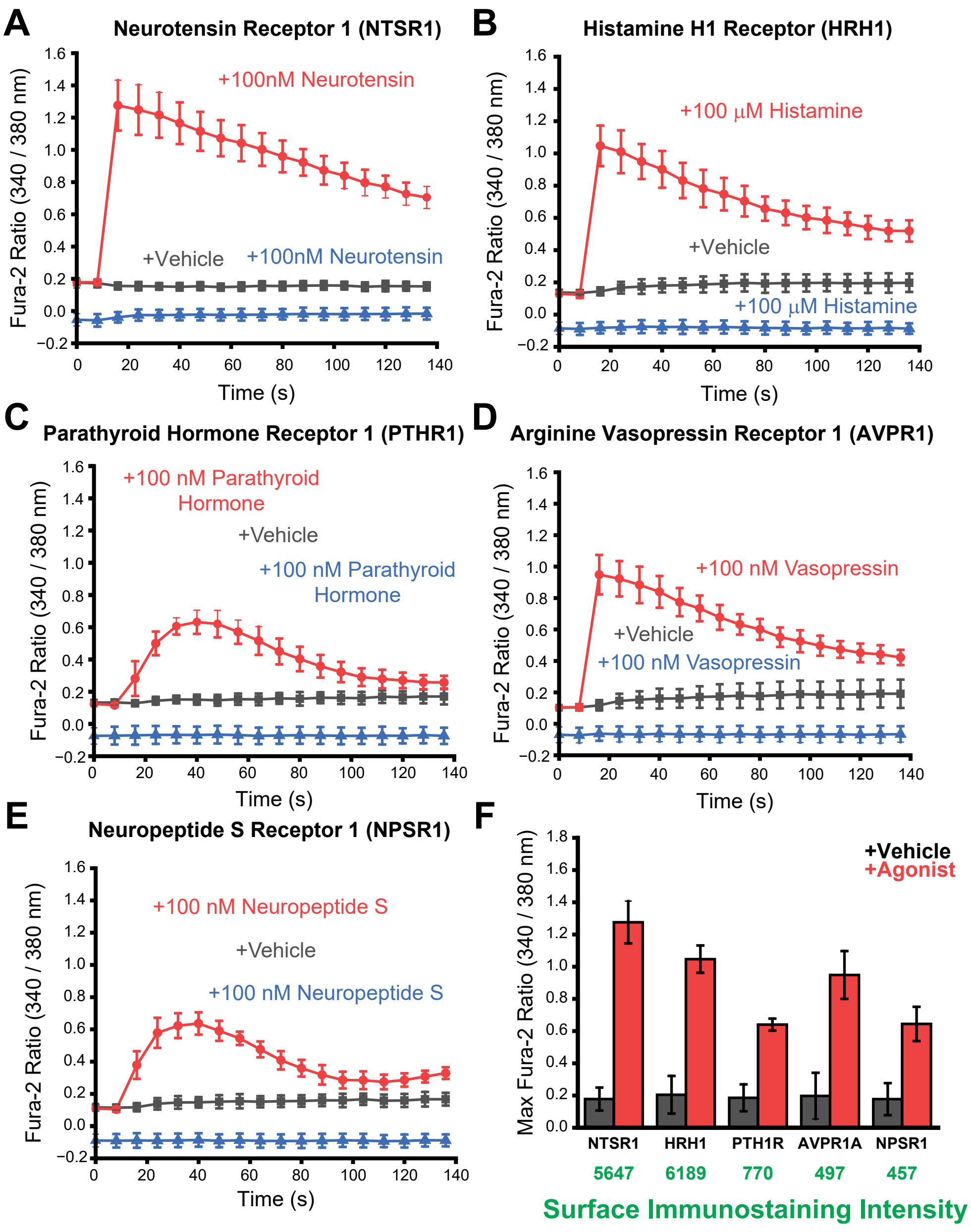

Figure S3. Signaling Activity of GPCRs with Divergent Plasma Membrane Expression. The signaling activity of five GPCRs with varying plasma membrane expression was compared in the context of recombinent HEK293T cells by monitoring calcium fluxes generated in the presence of agonists. A series of recombinant HEK293T cell lines expressing A) NTSR1, B) HRH1, C) PTHR1, D) AVPR1, or E) NPSR1 were incubated with a calcium-sensitive Fura-2 dye prior to introduction of agonists. A series of line plots depict the change in the Fura-2 fluorescence emission ratio over time after the addition of agonist (red) or vehicle (gray) to GPCR-expressing cells or addition of agonist to mock transfected cells (blue). F) A bar plot depicts the peak emission ratio achieved for cells expressing each receptor under each condition. The corresponding surface immunostaining intensities of each receptor by deep receptor scanning is shown in green for reference. Emission ratio values reflect the average of nine technical replicates collected across three biological replicates and error bars reflect the standard deviation. We note that the transcripts of these receptors were not observed within RNA-Seq measurements of the parental cell line. Together, these results demonstrate the the plasma membrane expression of recombinantly expressed GPCRs does not limit signal transduction in the context of this cell line.

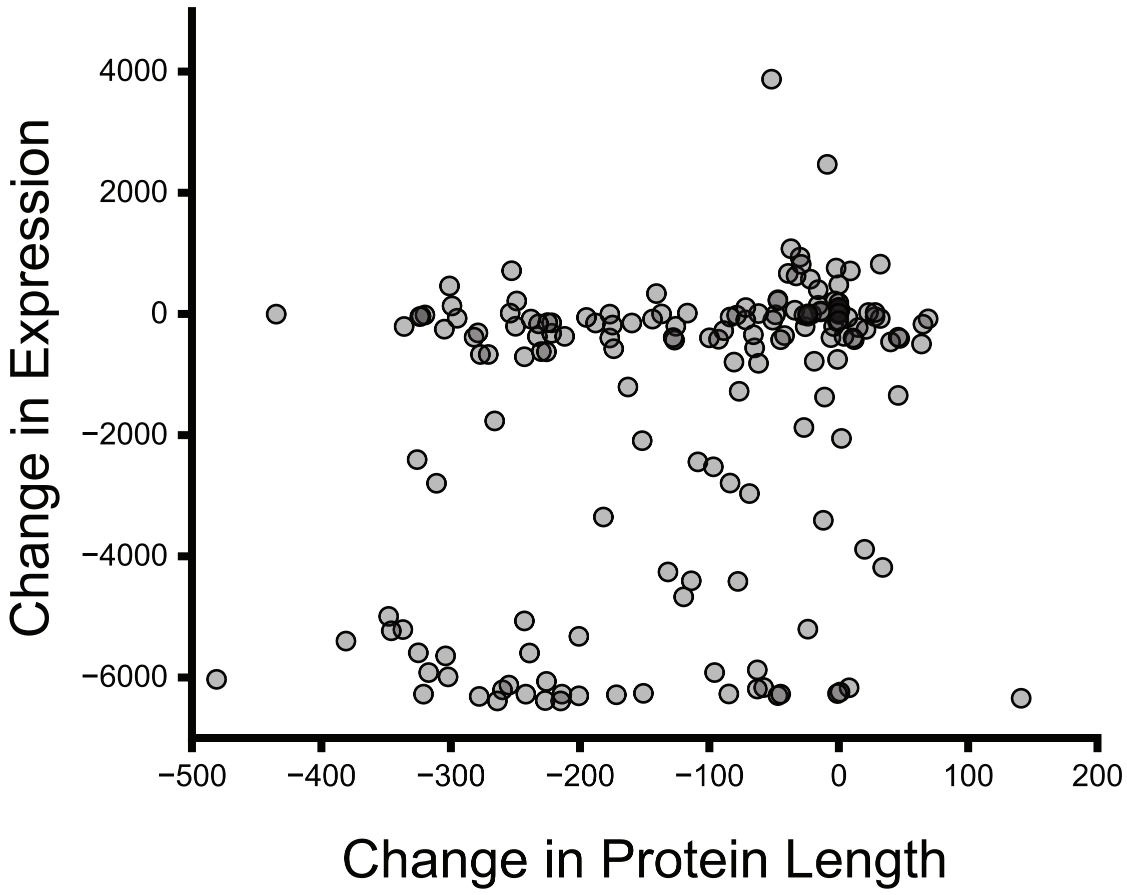

Figure S4. Trends in the Plasma Membrane Expression of GPCR isoforms with Varying Length. A Scatterplot depicts the change in the plasma membrane expression of each isoform in relation to its corresponding change in protein length according to deep receptor scanning measurments. Changes in expression and length are cast in terms of the isoform receptor value minus the cannonical receptor value.

Table S1. Supervised Model Performance

Models trained to classify the full set of GPCRs (n= 781) w/ the full feature set (n= 72).*

Model	Balanced Accuracy	F1-Score	Precision	Recall	ROC-AUC
Random Forest	0.731	0.532	0.438	0.677	0.810
XG Boost	0.653	0.454	0.590	0.370	0.841
Gradient Boosting	0.596	0.336	0.455	0.272	0.828

Models trained to classify GPCRs with stable transcripts (n= 452) w/ topological and structural features (n= 40).*

Model	Balanced Accuracy	F1-Score	Precision	Recall	ROC-AUC
Random Forest	0.735	0.646	0.600	0.700	0.784
XG Boost	0.645	0.510	0.564	0.471	0.731
Gradient Boosting	0.617	0.457	0.538	0.403	0.710

Models trained to classify GPCRs with stable transcripts (n= 452) w/ topological features (n= 27).*

Model	Balanced Accuracy	F1-Score	Precision	Recall	ROC-AUC
Random Forest	0.600	0.415	0.559	0.337	0.693
XG Boost	0.624	0.468	0.555	0.422	0.704
Gradient Boosting	0.603	0.445	0.524	0.413	0.696

Models trained to classify GPCRs with stable transcripts (n= 452) w/ structural features (n= 13).*

Model	Balanced Accuracy	F1-Score	Precision	Recall	ROC-AUC
Random Forest	0.586	0.441	0.448	0.433	0.654
XG Boost	0.575	0.420	0.451	0.403	0.641
Gradient Boosting	0.553	0.357	0.439	0.318	0.644

**Certain isoforms lack seven transmembrane domains and do not contain a full set of topological features. For this reason, they were excluded from model training. GPCRs we considered to be encoded by stable transcripts if recombinant cells expressing these cells were not statistically enriched within the sub-population of low-GFP cells.*

Table S2. Relative Contribution of Features in Different Models

Rank	All GPCRs All Features		High-GFP GPCRs Structure + Topology		High-GFP GPCRs Topology Features		High-GFP GPCRs Structural Features	
	Feature	Feature Importance	Feature	Feature Importance	Feature	Feature Importance	Feature	Feature Importance
1	Charged SASA	0.06885	$\Delta G_{app,pred}$ TMD6	0.080533	$\Delta G_{app,pred}$ TMD6	0.099171	Total SASA	0.113689
2	Total SASA	0.063461	Total SASA	0.048065	C-term Loop Length	0.063678	isolated beta bridge	0.092045
3	sasa aplr	0.044372	N-term loop length	0.042465	N-term Loop Length	0.056413	turn	0.091883
4	$\Delta G_{app,pred}$ TMD6	0.041741	ECL2 Length	0.040885	Molecular weight	0.050984	Charged SASA	0.09081
5	coil	0.037984	C-term Loop Length	0.03667	ECL2 Length	0.050774	3_{10} Helix Content	0.080018
6	Molecular weight	0.033951	turn	0.036154	TMD3 Length	0.043721	coil	0.077969
7	ECL2 Length	0.032032	3_{10} Helix Content	0.03605	TMD5 Length	0.042167	sasa aplr	0.074445
8	TMD3 Length	0.031234	Molecular weight	0.034632	$\Delta G_{app,pred}$ TMD2	0.04085	Alpha Helix	0.07385
9	Alpha Helix	0.030254	Charged SASA	0.033908	$\Delta G_{app,pred}$ TMD4	0.039665	f_crg	0.066522
10	C-term loop length	0.028549	sasa aplr	0.025969	TMD1 Length	0.033791	sasa_plr	0.065592
11	isolated beta bridge	0.026309	instability_index	0.02442	Aromaticity	0.032786	extended_configuration	0.06172
12	N-term loop length	0.022469	$\Delta G_{app,pred}$ TMD2	0.023666	ECL 3	0.032244	f_aplr	0.056127
13	sasa_plr	0.022323	Alpha Helix	0.023285	gravy	0.032187	f_plr	0.055329
14	gravy	0.021966	$\Delta G_{app,pred}$ TMD4	0.022707	TMD2 Length	0.032046		
15	TMD1 Length	0.021778	coil	0.022491	instability_index	0.031132		
16	3_{10} Helix Content	0.018651	Isoelectric_Point	0.022379	Isoelectric_Point	0.030519		
17	TMD6 Length	0.016432	Length TMD5	0.021388	$\Delta G_{app,pred}$ TMD1	0.029213		
18	TMD5 Length	0.016409	isolated beta bridge	0.021379	$\Delta G_{app,pred}$ TMD7	0.028785		
19	$\Delta G_{app,pred}$ TMD4	0.014686	TMD3 Length	0.021124	TMD6 Length	0.028595		
20	TMD2 Length	0.014626	gravy	0.02077	ICL3 Length	0.02731		
21	ICL1 Length	0.014592	Aromaticity	0.02071	ECL1 Length	0.026695		
22	turn	0.014479	TMD2 Length	0.020583	TMD4 Length	0.025685		
23	extended_configuration	0.013478	ICL2 Length	0.020281	ICL2 Length	0.024597		
24	Aromaticity	0.01305	ECL3 Length	0.02024	ICL1 Length	0.024527		
25	minRNAss	0.012463	$\Delta G_{app,pred}$ TMD5	0.019679	$\Delta G_{app,pred}$ TMD3	0.024473		
26	TMD4 Length	0.012326	TMD1 Length	0.0195	TMD7 Length	0.024021		
27	instability_index	0.012162	ICL1 Length	0.019099	$\Delta G_{app,pred}$ TMD5	0.023968		
28	ICL3 Length	0.012093	f_crg	0.018957				

29	$\Delta G_{app,pred}$ TMD2	0.011758	sasa_plr	0.018542				
30	$\Delta G_{app,pred}$ TMD7	0.010102	f_aplr	0.017918				
31	X40freqens	0.009905	$\Delta G_{app,pred}$ TMD3	0.017857				
32	ICL3 Length	0.009555	ICL3 Length	0.017462				
33	$\Delta G_{app,pred}$ TMD3	0.009227	TMD7 Length	0.017461				
34	zeroto38q25RNAss	0.008998	TMD6 Length	0.017086				
35	ECL1 Length	0.008905	extended_configuration	0.016663				
36	$\Delta G_{app,pred}$ TMD1	0.008561	$\Delta G_{app,pred}$ TMD1	0.016523				
37	ECL2 Length	0.008498	f_plr	0.015895				
38	Isoelectric_Point	0.008326	$\Delta G_{app,pred}$ TMD7	0.01587				
39	maxRNAss	0.008219	TMD4 Length	0.015527				
40	zeroto38minRNAss	0.007767	ECL1 Length	0.015207				
41	X40deltaG	0.007654						
42	zeroto38maxRNAss	0.007625						
43	zeroto38avgRNAss	0.007579						
44	$\Delta G_{app,pred}$ TMD5	0.007501						
45	TMD7 Length	0.006926						
46	q25RNAss	0.006914						
47	tAI10Min	0.006903						
48	tAI10q25.25.	0.006782						
49	plus10valRNAss	0.006613						
50	CpG_frame1_2	0.006416						
51	avgCU_first5	0.005919						
52	avgCU_first10	0.0059						
53	avgRNAss	0.005713						
54	GC10min	0.005621						
55	q75RNAss	0.005361						
56	tAI10q75.75.	0.005278						
57	avgCU	0.005229						
58	Nc	0.005097						
59	CAI	0.005036						
60	freqens	0.005031						
61	zeroto38q75RNAss	0.004906						

62	deltaG	0.00482						
63	CPSpL	0.004742						
64	avgCU_first 20	0.004732						
65	CPS_sum	0.00466						
66	GC10max	0.004538						
67	GC10q75	0.004172						
68	GC10q25	0.00414						
69	Global_tAI	0.004113						
70	GC3s	0.003927						
71	tAI10Max	0.00391						
72	GC	0.003705						

Table S3. Overview of Machine Learning Features

Transcript Feature Name	Description
CAI	Codon Adaptation Index. Category: Overall Codon Usage. Calculation Method/Tool: codonW 1.4.4
Nc	Effective number of codons. Category: Overall Codon Usage. Calculation Method/Tool: codonW 1.4.4
GC3s	GC content at the synonymous position. Category: Nucleotide. Calculation Method/Tool: Custom
CpG_frame1_2	Frequency of CG di-nucleotides. Category: Nucleotide. Calculation Method/Tool: Custom
avgCU	Average Codon Usage. Category: 5' Codon Usage. Calculation Method/Tool: Biopython
CPS_sum	Sum of Codon Pair Score values. Category: Codon Pair Score. Calculation Method/Tool: Coleman et al., 2008
CPSpL	Codon Pair Bias. Category: Codon Pair Score. Calculation Method/Tool: Coleman et al., 2008
Global_tAI	tRNA Adaptation Index. Category: tRNA Adaptation Index. Calculation Method/Tool: codonR
tAI10Min	Minimum tAI score over 10 codon windows. Category: tRNA Adaptation Index. Calculation Method/Tool: codonR
tAI10Max	Maximum tAI score over 10 codon windows. Category: tRNA Adaptation Index. Calculation Method/Tool: codonR
TAI10q25.25.	25th percentile of tAI scores over 10 codon windows. Category: tRNA Adaptation Index. Calculation Method/Tool: codonR
TAI10q75.75.	75th percentile of tAI scores over 10 codon windows. Category: tRNA Adaptation Index. Calculation Method/Tool: codonR
avgCU_first20	Codon Usage over the first 20 codons. Category: 5' Codon Usage. Calculation Method/Tool: Biopython
avgCU_first5	Codon Usage over the first 5 codons. Category: 5' Codon Usage. Calculation Method/Tool: Biopython
avgCU_first10	Codon Usage over the first 10 codons. Category: 5' Codon Usage. Calculation Method/Tool: Biopython
GC	Overall GC content. Category: Nucleotide. Calculation Method/Tool: Custom
GC10min	Minimum %GC over 10 codon windows. Category: Nucleotide. Calculation Method/Tool: Custom
GC10q25	25th percentile of %GC over 10 codon windows. Category: Nucleotide. Calculation Method/Tool: Custom
GC10q75	75th percentile of %GC over 10 codon windows. Category: Nucleotide. Calculation Method/Tool: Custom
GC10max	Maximum %GC over 10 codon windows. Category: Nucleotide. Calculation Method/Tool: Custom
X40deltaG	ŒîG of the lowest free energy structure for the first 40 codons. Category: 5' RNA Structure. Calculation Method/Tool: RNAfold
X40freqens	Frequency of the lowest free energy structure within the ensemble for the first 40 codons. Category: 5' RNA Structure. Calculation Method/Tool: RNAfold
plus10valRNAss	Average hybridization probability centered around +10 base, i.e. average of +5 to +15. Category: 5' RNA Structure. Calculation Method/Tool: RNAfold
zeroto38avgRNAss	Average hybridization probability over 10 base windows from 0 to +38. Category: 5' RNA Structure. Calculation Method/Tool: NUPACK

zeroto38minRNAss	Minimum hybridization probability over 10 base windows from 0 to +38. Category: 5' RNA Structure. Calculation Method/Tool: NUPACK
zeroto38q25RNAss	25th percentile of hybridization probability over 10 base windows from 0 to +38. Category: 5' RNA Structure. Calculation Method/Tool: NUPACK
zeroto38q75RNAss	75th percentile of hybridization probability over 10 base windows from 0 to +38. Category: 5' RNA Structure. Calculation Method/Tool: NUPACK
zeroto38maxRNAss	Maximum hybridization probability over 10 base windows from 0 to +38. Category: 5' RNA Structure. Calculation Method/Tool: NUPACK
deltaG	ŒîG of the lowest free energy structure. Category: Overall RNA Structure. Calculation Method/Tool: RNAfold
freqens	Frequency of the lowest free energy structure within the ensemble. Category: Overall RNA Structure. Calculation Method/Tool: RNAfold
avgRNAss	Average hybridization probability over 10 base windows. Category: Overall RNA Structure. Calculation Method/Tool: NUPACK
minRNAss	Minimum hybridization probability over 10 base windows. Category: Overall RNA Structure. Calculation Method/Tool: NUPACK
q25RNAss	25th percentile of hybridization probability over 10 base windows. Category: Overall RNA Structure. Calculation Method/Tool: NUPACK
q75RNAss	75th percentile of hybridization probability over 10 base windows. Category: Overall RNA Structure. Calculation Method/Tool: NUPACK
maxRNAss	Maximum hybridization probability over 10 base windows. Category: Overall RNA Structure. Calculation Method/Tool: NUPACK

Protein Feature name	Description
Length_without_SP	Length of the protein after removal of the signal peptide.
TMD1-7 Length	Normalized lengths of each transmembrane domain (TMD1–TMD7), relative to the full-length protein. TMDs are predicted using **DeepTMHMM**, and their coordinates are refined using the **von Heijne transfer free energy scale** von_heijne_scan_functions_v39_signal_pep_topcons.py (https://github.com/schebachlab/PRF-Search).<br>The lengths are then normalized to the length of protein
N-term Loop Length	Normalized length of the N-terminal extracellular region preceding TMD1.
ICL1-3 Length	Normalized lengths of the intracellular loops immediately following TMD1, TMD2, and TMD3, respectively.
ECL1-3 Length	Normalized lengths of the extracellular loops immediately preceding TMD2, TMD3, and TMD4, respectively.
C-term Loop Length	Normalized length of the C-terminal intracellular region following TMD7.
$\Delta G_{app,pred}$ TMD1-7	Free energy of membrane insertion (ΔGAPP,PRED) for each TMD (TMD1–TMD7), computed using the **von Heijne transfer free energy scale** via von_heijne_scan_functions_v39_signal_pep_topcons.py (https://github.com/schebachlab/PRF-Search). These ΔGAPP,PRED values reflect the biophysical favorability of each TMD's insertion into the membrane.
Molecular_Weight	The molecular weight of the protein (in Daltons), computed from its amino acid composition.
Isoelectric_Point	The theoretical pH at which the protein carries no net charge.
Aromaticity	The fraction of aromatic amino acids (Phe, Trp, Tyr) present in the protein.
instability_index	Predicts the in vitro stability of a protein based on dipeptide composition. Values greater<br>than 40 suggest stability; values less 40 indicate instability. Defined by Guruprasad et<br>al. (1990):(https://pubmed.ncbi.nlm.nih.gov/2075190/)
gravy	Average hydrophobicity of the amino acids in the sequence using the Kyte–Doolittle<br>scale: https://doi.org/10.1016/0022-2836(82)90515-0.

Total SASA	The total solvent-accessible surface area (SASA) of the protein, calculated using FreeSASA:<br>https://freesasa.github.io/.
sasa_crg<br>sasa_plr<br>sasa_aplr	Contributions to total SASA from:<br>– Charged SASA: Charged residues (Asp, Glu, Lys, Arg,His).<br>– SASA plr: Polar residues (Ser, Thr, Asn, Gln, Tyr, Cys).<br>– SASA aplr: Apolar (non-polar) residues (Ala, Val, Ile, Leu, Met, Phe, Trp, Pro,<br>Gly).
f_crg<br>f_plr<br>f_aplr	Fractions of the total SASA contributed by charged, polar, and apolar residues, respectively<br>(range: 0–1).
alpha_helix	Fraction of residues in alpha-helical conformation, as assigned by STRIDE.
3_{10} Helix Content	Fraction of residues in 3_{10} helix conformation.
extended_configuration	Fraction of residues in extended strand (beta-sheet-like) conformations.
isolated_beta_bridge	Fraction of residues forming isolated beta-bridges (not part of extended beta-sheets).
turn	Fraction of residues in turns—tight loops reversing the direction of the peptide backbone.
coil	Fraction of residues in unordered, coil-like conformations.
